# The influence of spatiotemporal conditions and personality on survival in reintroductions–evolutionary implications

**DOI:** 10.1007/s00442-016-3740-0

**Published:** 2016-10-08

**Authors:** Marianne Haage, Tiit Maran, Ulrika Alm Bergvall, Bodil Elmhagen, Anders Angerbjörn

**Affiliations:** 1 Department of Zoology, Stockholm University, 106 91 Stockholm, Sweden; 2 Conservation Research Lab, Tallinn Zoological Gardens, Paldiski Mnt.145, Tallinn, 13522 Estonia; 3 Institute of Veterinary Medicine and Animal Sciences, Estonian University of Life Sciences, Kreutzwaldi 62, 51014 Tartu, Estonia

**Keywords:** Fitness, *Mustela lutreola*, Reintroduction, Spatiotemporal variation, Radio-tracking

## Abstract

Personality exists in non-human animals and can impact fitness. There is, however, a shortage of empirical studies in certain areas within the field, and fundamental evolutionary theory on personality remains largely untested. For example, little is known on how variation in personality is maintained over evolutionary time. Theory suggests that fluctuating selection pressures due to spatiotemporal variation in conditions, e.g. food availability, is a possible mechanism and a few studies have shown that the success of different personality types varies with spatiotemporal conditions. However, it remains unknown whether different mechanisms can maintain personality within a species. Here we use a reintroduction programme for the critically endangered European mink (*Mustela lutreola*) to test whether multiple personality trait domains (boldness, exploration and sociability) affected survival in two different years and islands. This was done through pre-release personality tests and post-release radio-tracking monitoring. Survival was positively correlated with boldness, whereas the relationship with exploration was either negative or positive depending on year/island. The results show a complex relationship between personality and survival and suggest that exploration can be maintained over evolutionary time via spatiotemporal variation in conditions. However, in contrast to exploration, boldness did not vary spatiotemporally and sociability had no impact on survival. This indicates that different personality trait domains might be maintained by different mechanisms. To date, personality has been studied primarily within behavioural sciences, but through empirical findings we highlight the importance of personality also in ecology and conservation biology.

**Electronic supplementary material:**

The online version of this article (doi:10.1007/s00442-016-3740-0) contains supplementary material, which is available to authorized users.

## Introduction

In recent years, individual variation has received increasing attention as it can explain non-optimality and noise in data sets (Wolf and Weissing 2012; Sih et al. 2012). It occurs in several traits, such as morphology and behaviour, but individual variation in animal personality is currently one of the fastest growing fields in biology. Animal personality is defined as behavioural differences between and within individuals, repeatable over time, situations or contexts (Sih et al. 2004; Réale et al. 2007), and it has been confirmed in numerous species (Gosling 2001). Similar terms include behavioural syndromes, temperament and copying styles. Personality can be divided into groups of correlated behaviours called personality trait domains, for example, boldness (risk-taking), exploration (exploratory behaviour in novel environments) and sociability (non-antagonistic behaviours towards conspecifics; Sih et al. 2004; Réale et al. 2007). However, although there is theoretical literature on the selection of personality (e.g. Wolf et al. 2007; Wolf and Weissing 2010), there is a need for further empirical and hypothesis-driven studies on animal personality (Dall and Griffith 2014). Such studies are necessary to understand how personalities have evolved and are maintained in natural populations over evolutionary time.

To investigate evolutionary aspects of personality, one must determine whether personalities are adaptive. There is evidence for genetic correlations with personality trait domains (Taylor et al. 2012; Johnson et al. 2015), and personalities are at least partially heritable and impacted by additive genetic variation (Fairbanks et al. 2004; van Oers et al. 2005; Rogers et al. 2008; Taylor et al. 2012; Fawcett et al. 2014; Johnson et al. 2015). Many studies also show a relationship between personality and fitness. For example, meta-analyses show that high boldness has a positive impact on mating success in captive/domesticated animals but a negative impact on the life-span of wild animals (Smith and Blumstein 2008). In captivity long-term studies on neuroticism, agreeableness, dominance and extraversion in gorillas (*Gorilla gorilla gorilla*) show that extraversion positively related to lifespan (Weiss et al. 2013).

Although we can assume personality to be adaptive, the mechanisms maintaining variation in personality within populations are poorly understood. Several mechanisms have been suggested, for example, frequency dependent selection and spatiotemporal variation in conditions (Wolf and Weissing 2010). Spatiotemporal variation in conditions can cause fluctuating selection pressures which may favour different personality types at different times/places, and thus maintain different personality types within populations over time (Wolf and Weissing 2010). However, the empirical support for this mechanism is limited to a few studies. In great tits (*Parus major*), both adult and offspring survival were affected by behaviour in novel environments, and the success of different personality types varied between years, perhaps due to fluctuating food availability (Dingemanse et al. 2004). Likewise, in North American red squirrels (*Tamiasciurus hudsonicus*) the impact of female aggression and activity on measures of reproductive success (juvenile survival in the nest and during their first winter and growth rate) varied between years, probably also caused by fluctuating food availability (Boon et al. 2007). Still it remains unknown whether fluctuating spatiotemporal conditions are a major mechanism for maintaining personality over time, and whether the mechanism maintaining personality differs between personality trait domains.

Personality differences between individuals should be repeatable over situations and contexts (Sih et al. 2004; Réale et al. 2007), and personality expressed in captivity can mirror personality expressed in the wild (Herborn et al. 2010). Reintroduction programmes for endangered animal species, hence, present a model system where influences of personality on fitness can be studied under different spatiotemporal conditions. First, individual variation in personality can be experimentally tested in captivity, providing an opportunity to properly assess personality trait domains. Second, all animals can be raised under the same controlled conditions. Third, the participating individuals can be chosen based on their personality, so that a wide spectrum of personality types is represented in the study. This can be difficult in wild-trapped animals as bold individuals tend to be easier to trap than shy individuals (e.g. Carter et al. 2012b). Studies on endangered species also have applied perspectives, as reintroduction programmes often have high mortality rates (Letty et al. 2007). Knowledge on personality impacts on survival could thus improve conservation methodology (Watters and Meehan 2007). However, to our knowledge, there is only one scientific study on reintroductions where individual variation in personality has been considered. Bold swift foxes (*Vulpes velox*) had a higher reproductive success in captivity than shy individuals, but also a higher risk of early death after reintroduction (Bremner-Harrison et al. 2004). Since the influence of personality on survival seems to be sensitive to spatiotemporal conditions (Dingemanse et al. 2004; Boon et al. 2007) understanding of this relationship is also important to implement a personality perspective with species conservation.

In this study, we use an Estonian reintroduction programme for the critically endangered European mink (*Mustela lutreola*) as model system. Predation has been the primary cause of death for animals following release, and females have had a lower survival rate than males (Maran et al. 2009). However, the probability of survival has not been affected by movement patterns (Harrington et al. 2014), number of preceding generations in captivity for each individual, release method, type of housing before release or age (Maran et al. 2009). Moreover, released European mink can adapt to a natural diet. During 60 days post-release natural prey species are caught increasingly (1.5–3 times) whilst the use of atypical food sources decline fivefold (Põdra et al. 2013). Hence, we hypothesise that personality may explain individual variation in survival, but the relationship may vary with spatiotemporal conditions.

To test this hypothesis, the animals were first tested in captivity for boldness, sociability and exploration, which are repeatable personality trait domains in European mink (Haage et al. 2013). The subsequent reintroduction was then used as a field experiment, testing whether these personality traits affected survival during initial dispersal and establishment, which is a prerequisite for future reproduction, and thus overall fitness. We predict that boldness, i.e. the level of risk-taking behaviour, and exploration, i.e. behaviour related to the exploration of new areas, are more likely to affect survival than sociability. Sociability, i.e. non-antagonistic behaviour towards conspecifics, should not affect survival as the European mink is strictly solitary-living outside the breeding season. We also tested whether the relationship between personality and survival was influenced by spatiotemporal conditions, as animals were released in two different years and islands, and discuss how variation in spatiotemporal conditions may act to maintain personality in European mink.

## Methods

### Study species

The European mink is a semi-aquatic generalist carnivore inhabiting brooks, rivers and wetlands. It is solitarily living, except during mating and offspring-rearing. The breeding season is in March to April and the species is polyestrous. After 42 days of gestation litters are born in May to June. Dispersal occurs when the animals are fully grown, at the age of 2.5–4 months, where after territories are established (Youngman 1990; Amstislavsky et al. 2009; Maran et al., 2009; Nagl et al. 2015). The European mink is extinct in most parts of its former distribution area. It is classified as critically endangered on the IUCN red list (IUCN 2014) and listed in Appendix II of the Bern Convention. The major causes of decline are hunting, competition from the invasive American mink (*Neovison vison*) and habitat loss (Maran and Henttonen 1995; Maran et al. 1998; Maran 2007).

### Housing

The animals in this study were born in captivity in an off-public conservation breeding facility at Tallinn Zoological Gardens in Estonia. They were housed in two different types of outdoor enclosures; one smaller standard type and one larger type, mainly intended for soft release purposes. Soft release involves raising animals in an environment similar to the release site to give them opportunity to acquire skills needed for survival in the wild and, hence, make the transition from captivity as smooth as possible (for examples see Bright and Morris 1994; de Milliano et al. 2016). The standard enclosures (200 × 400 × 180 cm) contained water for swimming (64 × 35 × 30 cm), tunnels and various vegetation and natural objects such as roots and branches on a sand and dirt floor. In connection to the enclosures there was a nest box divided into two compartments (34 × 25 × 27 cm each) whereof one was filled with straw. The larger enclosures (25–50 m^2^) had natural vegetation and ground cover, ponds (1.5 × 1.5 × 1.0 m–2.5 × 2.5 × 1.0 m) and the same type of nest boxes placed on the ground. The standard enclosures were cleaned daily and all animals were fed once per day with rodents, fish, birds or minced meat with added vitamins. There were no predation-stimuli in the enclosures. To avoid habituation to humans, contact with humans was minimized.

All animals used in the reintroduction study were born in large enclosures approximately 3 months prior to their release, with the exception of two adult males (Table 1). The two adult males, which were born and held in standard enclosures, were included in the study due to a shortage of males in the litters of the years of 2012 and 2013. A previous study showed that post-release survival is not affected by age (Maran et al. 2009), and preliminary analyses showed that exclusion of the two adults did not affect the results. The animals born in large enclosures were moved into standard enclosures 5–7 days prior to the personality testing since it was not possible to effectively catch them and perform experiments in the large enclosures. In addition to the reintroduced animals, 19 animals born and held in standard enclosures were used for method verification regarding personality tests.

**Table 1:** Details and fates of radio-tracked European minks reintroduced in Estonia

Sex	Release year	Age	Enclosure size*	Status^†^	Days alive	Assessed cause of death
f	2012	0	Large	Dead	2	Predation
m	2012	5	Small	Lost		–
f	2012	0	Large	Dead	35	Predation
f	2012	0	Large	Dead	2	Predation
f	2012	0	Large	Dead	32	Predation
m	2012	0	Large	Dead	3	Predation
m	2012	6	Small	Dead	18	Road killed
f	2012	0	Large	Dead	3	Predation
f	2012	0	Large	Dead	12	Starvation
f	2012	0	Large	Dead	24	Predation
m	2013	0	Large	Alive	60	–
m	2013	0	Large	Lost		–
f	2013	0	Large	Dead	3	Predation
m	2013	0	Large	Alive	60	–
f	2013	0	Large	Alive	60	–
f	2013	0	Large	Alive	60	–
m	2013	0	Large	Alive	60	–
f	2013	0	Large	Lost		–
f	2013	0	Large	Dead	35	Predation
f	2013	0	Large	Lost		–
f	2013	0	Large	Alive	60	–
m	2013	0	Large	Lost		–
m	2013	0	Large	Alive	60	–
f	2013	0	Large	Dead	25	Predation
f	2013	0	Large	Unknown	Unknown	Unknown

In 2012 animals were released on the island Saaremaa (*N* = 10) and in 2013 animals were released on the neighbouring island Hiiumaa (*N* = 15). *N*_total_ = 25; *N*_female_ = 16; *N*_male_ = 9. ‘Days alive’ shows the number of days animals were alive after release, with a maximum of 60 days. After 60 days animals were live-trapped for collar removal and capture time varied. The fate of one animal has been labelled as unknown as a mortality signal was retrieved from an underground drainage tunnel which could not be accessed (please see “Results” for details). Lost animals include those which lost their collars (i.e. only the collar was found and there were no signs of predation), ventured into inaccessible land areas on the islands or had collars subject to technical malfunction

* All animals raised in large enclosures were transferred to small enclosures 5–7 days before the start of the personality tests prior to the release. The large enclosures were 25–50 m^2^ and the small enclosures were 8 m^2^, see “Methods” for further information

^†^At the end of the radio-tracking period of 60 days

### Pre-release personality testing

Haage et al. (2013) identified three personality trait domains in European mink; boldness, exploration and sociability. The domains (and also single behaviours), were repeatable within and between situations (repeatability = 0.40–0.69). Single behaviours were measured and scored in a number of different tests and contexts (described below), and subsequently standardized and analysed statistically with principal component analysis (PCA) to identify groups of correlated behaviours, i.e. personality trait domains (Table 2). This means that the naming of the domains were assessments based on multiple tests. There are several advantages with this approach. For example, the structure of domains can be seen in high resolution and it minimises the risk of so called jingle fallacies (see Block 1995), i.e. failure to separate between traits, which might happen if a test is assumed to measure a certain personality trait domain when it in fact measures something else (for a full discussion on this subject, see Haage et al. 2013). Furthermore, it is likely most informative to include several personality trait domains when investigating personality and fitness (Carter et al. 2012a; Haage et al. 2013).

**Table 2:** Principal component analysis table from Haage et al. (2013) showing the structure of personality in European mink based on single behaviours measured in different tests and test contexts

Experiment	Measure	Boldness	Sociability	Exploration
Novel object in home enclosure	Latency^a^	**−0.860**	−0.087	−0.175
	Approach^b^	**0.875**	0.081	0.170
	Sniff^c^	**0.821**	0.058	0.155
	Attack/bite object	**0.731**	−0.112	0.025
	Carry object away	**0.761**	0.053	0.047
Mirror image stimuli in home enclosure	Latency^a^	**−0.660**	−0.321	0.304
	Look^d^	**0.626**	0.439	−0.316
	Sniff^c^	**0.636**	0.396	−0.311
	Push/dig/scratch^e^	**0.559**	0.229	−0.307
	Attack/bite mirror image	**0.438**	−0.117	−0.079
Mirror image stimuli in novel arena	Latency^a^	−0.097	−**0.866**	−0.107
	Look^d^	0.048	**0.911**	0.076
	Sniff^c^	0.124	**0.895**	0.065
	Mark^f^	0.099	**0.811**	0.110
	Hiss	−0.161	0.276	−0.118
	Push/dig/scratch^e^	−0.031	0.210	**0.550**
	Attack/bite mirror image	0.013	0.136	**0.479**
Novel arena	Latency^a^	−0.257	−0.468	−**0.574**
	Zones visited^g^	0.153	0.462	**0.595**
	Hiss	0.010	0.088	**0.625**
	Mark^f^	0.075	0.369	0.336
	Explained variation	5.120	4.303	2.242
	Proportion of total	0.248	0.205	0.107

The components were rotated with the varimax procedure and include 21 behavioural measures collected in experiments with fully grown animals aged 0–6 years (*N*_female_ = 40; *N*_male_ = 40). The tests were performed in November and December 2009 (non-breeding season). Numbers in boldface indicate salient loadings (≥0.4). In case of several salient loadings in different components for one behaviour the highest value has been used as personality trait in the present paper

^a^Latency in seconds to leave the nest/transport box

^b^The body is positioned towards a novel object at a maximum distance of 20 cm

^c^Sniffing at a novel object or mirror image. See “Methods” for more information

^d^Looking at the mirror image. See “Methods” for more information

^e^Scratch, push or dig at the mirror image

^f^Visible markings including anal drags

^g^The number of zones visited in the novel arena (see “Methods” for more information)

The tests carried out by Haage et al. (2013) were (1) novel object tests which test the reaction toward previously unknown and potentially risk-containing stimuli, (2) a mirror stimulus test in the home enclosure to test the reactions towards conspecifics within the own territory, (3) a novel arena test to test how individuals respond to novel environments and (4) a mirror stimulus tests in an arena to test the response to conspecifics outside the own territory. In the novel object test a rubber dog toy was placed in the middle of the home enclosure. The measured behaviours consisted of latency to leave the nest box (in seconds (s), max. 240 s, the same in all tests); approaching the object (the nose had to be ≤20 cm from the novel object with the body positioned towards the object); sniffing on the object; attacking or biting the object; carrying away the object. The test was repeated within both non-breeding and breeding season (with different novel objects) to measure repeatability. In the mirror stimulus test in the home enclosure, a mirror was facing the nest box exit. The measured behaviours consisted of latency to leave the nest box; looking at the mirror image; sniffing at the mirror image; attacking or biting the mirror image; scratching, pushing or digging at the mirror image. In the novel arena test the animals were released from transport boxes into an arena (190 cm × 240 cm with a tile floor and plywood walls) marked in 20 cm wide circular zones. The measured behaviours consisted of latency to leave the transport box; entering a new zone (with at least both front paws and the head, excluding the zone closest to the wall, where the transport box was placed, as they would automatically enter this if entering the arena); doing visible markings/anal drags; hissing. In the mirror stimulus tests in the arena, animals were released from transport boxes into an arena (190 cm × 240 cm with a tile floor and plywood walls) with a mirror facing the transport box. The measured behaviours consisted of latency to leave the transport box; looking at the mirror image; sniffing at the mirror image; attacking or biting the mirror image; scratching, pushing or digging at the mirror image; doing visible markings/anal drags; hissing.

All tests were done before 16:00, when the animals were fed, and lasted 240 s. Latencies were measured in seconds while all other behaviours were scored. To add a temporal dimension, animals received a higher score the earlier a behaviour was exhibited during the 240 s test period; 3 points if it was done in the first 80 s, 2 points in the second period of 80 s and 1 point in the last period of 80 s. To avoid unbalanced scores, the behaviours were only scored the first time they were made during a trial. Sniffing and looking behaviours were divided into more or less than 1 s, depending on how long they lasted, to separate hesitant and confident approaches. Subsequently, the two scores were weighted into one score by first doubling the score for the longer duration (to maintain definition on confident and hesitant approaches) and then adding both scores (Haage et al. 2013).

This method to assess personality traits in European mink showed (1) that a selection of tests would be sufficient to measure boldness, sociability and exploration, and (2) that these personality traits were repeatable both within and between situations for whole tests, domains and single behaviours (Haage et al. 2013). Minimising the number of tests can decrease potential stress for the animals, as well as the risk of habituation towards humans which is not desirable in reintroduced animals. Hence, in this study, we did not repeat tests as repeatability already had been confirmed, and we only carried out a selection of tests which were sufficient to measure boldness, sociability and exploration according to (Haage et al. 2013; Table 2). More precisely, two tests measured boldness (novel object and mirror stimulus in the home enclosure), wherefore, only one of these tests was carried out here. Exploration and sociability were measured with the novel arena test and the mirror test in the arena.

To measure boldness a modified version of the novel object test was used. The modifications were necessary as the animals in this study had been transferred to standard enclosures only 5–7 days before the tests. This time span was not enough to fully habituate to the new environment, and the dog toy presented as a novel object could thus be perceived as a part of the novel environment. Furthermore, due to the soft release intent with the large enclosures, we did not wish to hold the animals in standard enclosures longer than necessary to carry out personality tests. In the modified test, a human replaced the dog toy as novel object, as this was regarded to be a stronger stimulus that should present novelty and risk to the human-naive animals. To verify that this test measured boldness the test was also performed on 19 animals that had been subject to the original novel object test (in 2009; Haage et al. 2013). Since the score of the human test correlated significantly to the score of the novel object test it was regarded as an adequate measure of boldness (Spearman rank order correlation: *ρ* = 0.60; *P* = 0.006; *N* = 19). In the human test, the observer (the same person for all animals) sat in one end of the enclosure. The animal was released from a transport box 1 m from the observer, with the exit facing away from the observer (as most animals were still in litters with their mothers it was not possible to release them individually from the nest box). Each trial lasted 4 min and both the nest box and transport box were open during the test. The 3 s immediately after exiting the transport box was not considered, as the animal may need some seconds to orientate itself. Animals were then scored for how close they came to the human by dividing the enclosure into three zones, the one furthest away giving one point, the intermediate giving two points and the closest giving three points. If the animal stayed within the transport box it did not receive any points. The animals were also scored for approaching the human, defined as being within 30 cm from the human with its body being directed towards the human, as well as for sniffing on the human and interacting in other ways, for example, by scratching, pushing with the nose, pulling on the clothes, or sitting or climbing on the human. The same scoring system as described above was employed. All tests were performed with one animal at the time (most animals still lived together in litters with their mothers) between the 12th and 21st of August in both 2012 and 2013.

### Post-release radio-tracking

After the personality experiments, animals were equipped with radio-collars. They were then kept at the breeding facility with the collars on for at least 2 days before release to ensure that the collars were functioning properly and that the animals did not react negatively to the collars. The animals were transported 4–5 h to the release sites in boxes (15 × 19 × 41 cm). In both years of the study, the animals were released in Estonia between the 25th of August and 4th of September. The release in 2012 was done on the island Saaremaa (2673 km^2^; Lat 58.41; Long 22.48) and the release in 2013 on the neighbouring island Hiiumaa (989 km^2^; Lat 58.89; Long 22.62; Table 1). There were no European mink previous to the reintroduction programme on either island, but yearly releases of European mink on Hiiumaa since 2000 had created a small but not yet viable population (Maran et al. 2009; IUCN 2014). On Saaremaa there had been no previous releases. Both islands were free from the invasive American mink and contained suitable riparian habitats for European mink. Suitability of sites was based on prey availability and access to hiding and denning places, for example, roots, shrubs and stone piles. Release sites on Hiiumaa were also chosen based on habitat availability on suitable sites for release, estimated in previous inventories of established European mink. Since reintroduced European mink tend to establish territories within 1–1.5 months (Maran et al. 2009) the animals were monitored by radio-tracking for 60 days. After the 60 days surviving animals were trapped for removal of the radio-collars and then re-released on the capture sites.

In total, 31 personality-tested animals from nine litters were released, and 25 of these were included in the field study and radio-tracked (*N*_2012_ = 10; *N*_2013_ = 15). The number of radio-tracked animals was determined by the required and available manpower and by the amount of radio-collars at our disposal. To get a broad representation of different types of animals, individuals were chosen for monitoring based on personality type in the first place and sex in the second place.

The radio-tracking equipment was manufactured by ATS (Advanced Telemetry Systems, U.S.A.) and consisted of three receivers (two R2000 and one R410) and transmitters (M1600) mounted on neoprene collars (total weight 11 g, ~1–2 % of the body weight). If the collars were motionless for more than 12 h the normal signal was replaced by a mortality signal. The locations of the animals were recorded up to nine times per 24 h, but the intensity was lowered if animals established territories. The more an animal moved the more intense was the monitoring. When an animal was found dead the cause of death was assessed by a veterinarian with previous experience of work with European mink. Injuries and general physical condition was examined and predators were identified by bite marks on bodies and collars. Some animals were fully autopsied. Cause of death was only used as an indicator of the main cause of death (Table 1) and not used in the statistical analyses. If an animal could no longer be found the individual was labelled as lost, which could be due to loss of collar, technical malfunction or dispersal into inaccessible land areas on the islands.

### Ethics/permissions

The reintroductions were part of an ongoing conservation project for European mink in Estonia. Animals have been released yearly since 2000 in accordance to the European mink action plan in Estonia (see Maran et al. 2009). According to Estonian law and EU-legislation, the personality tests in this study did not require any permission. Still, animal welfare was an important element of the study design. All 31 animals that were personality tested were reintroduced on the Islands of Saaremaa and Hiiumaa in Estonia. The 19 animals that were subject to one behavioural test to verify methods were housed and kept as usual after the testing.

### Statistics

In Haage et al. (2013) single behaviours from the experiments were analysed with principal component analysis (PCA; Table 2) to identify groups of correlated behaviours, i.e. personality trait domains. For each personality trait domain (assessed as boldness, sociability and exploration) a score was given to each individual based on the single behaviours that fell into that personality trait domain. The scores were weighted according to the principal component scores with +1 for positive values, −1 for negative values and 0 for non-salient behaviours. In this study, the individual scores for each personality trait domain were calculated and weighted in accordance to the methods and PCA in Haage et al. (2013), where latencies where always weighted negatively and other scores always positively. This PCA was used to see which single behaviours fell into which personality trait domain instead of making a new analysis since the sample size (*N* = 80) was considerably larger, which leads to a more reliable analysis (for details see Pre-release personality testing and Haage et al. 2013).

General linear models were built to analyse the impact of boldness, sociability, exploration, year and sex on the number of days the animals survived. Our data had normally distributed residuals (Shapiro-Wilks test: *p* = 0.32), and thus met the criteria for general linear models (see also Appendix 1). Model selection was done through backward stepwise removal of non-significant terms. In our analyses we used 60 days as maximal survival as we lack data on the entire life-span of all individuals. However, this will likely underestimate the slope of each potential relationship, which means that the results are conservative. Animals that were lost or had unknown fates were excluded from the analysis.

To test whether the relationship between personality traits and survival varied with spatiotemporal conditions, interactions with year/island were included for each personality trait domain. Our sample size was not very high which could lead to an over-fitted model if all our variables and interactions were analysed in the same model. However, since the three personality trait domains that we use here were identified as uncorrelated separate entities (Haage et al. 2013) they should not have interacting influences on survival and separate models can be run. Hence we did three models; one for each personality trait domain. All these models were built as follows: survival ~ personality trait domain X + island/year + sex + personality trait domain X × island/year.

For models with small sample sizes, it is generally preferred to reduce the number of variables. We did not include LitterID, body weight, age or interactions between personality and sex in the final models. Preliminary analyses showed no impact of LitterID (*N* = 9), body weight and interactions between sex and personality trait domains and as these non significant variables fell out early in model selection the variables were excluded from any further analyses. The insignificance of body weight may be related to the fact that captive reared animals generally are more or less overweight. Age was not included as we only used two older animals and a previous study showed that age had no impact on survival. However, young of the year are recommended for release since they may reproduce for more seasons than older animals (Maran et al. 2009). Furthermore, exclusion of the two older individuals did not change the outcome of the statistical models.

To illustrate cumulative survival in the two different years a multiple group Kaplan-Meier survival analysis was performed, with year as grouping variable. In survival analysis the relationship between events and time is analysed and the technique also allows for the inclusion of data that are partially unknown in the form of censored data points. Here lost individuals and survivors were treated as censored data points. All analyses in this study were done in STATISTICA version 12 and the significance level was set to 0.05.

## Results

**Fig. 1:**
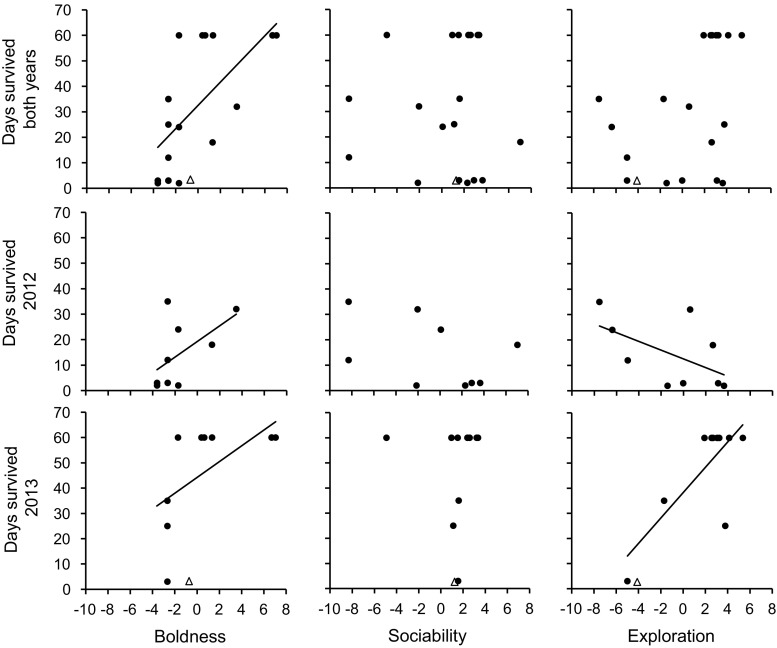
Relationships between individual scores for boldness, sociability and exploration in relation to days survived in European mink reintroduced in Estonia on the islands Saaremaa in 2012 and Hiiumaa in 2013. Trend lines are included for significant effects. Boldness had a positive effect on survival, sociability had no significant effect and the effect of exploration varied with year. *N*_total_ = 19; *N*_2012_ = 9; *N*_2013_ = 10. Animals that were lost or had an unknown fate were excluded from the statistical analysis and does thus not affect the trend lines presented in the graphs. However, as the death of one animal could not be fully confirmed albeit a persistent mortality signal was received (see “Results” for further details) this individual is presented as triangle shaped data point in the graphs for information purposes

The number of days that the animals survived during the 60 days of radio-tracking was positively related to boldness. Furthermore, there was a significant interaction between exploration and year, where exploration had a negative impact on survival in 2012 and positive in 2013. Year/island also affected survival directly in all three models, with lower survival in 2012 than 2013 (Fig. 1; see Table 3 for details and statistics).

**Table 3:** The impact of personality and release year/island on post-release survival (days survived) in radio-tracked European minks (*N* = 19) released on the Estonian islands Saaremaa in 2012 and Hiiumaa in 2013

Overall analysed model	Variable	Beta (*β*) ± SE	Partial eta-squared	Power	*p*	Adjusted *R* ^2^
Survival ~ boldness + island/year + sex + boldness × year/island	Year	−0.53 ± 0.15	0.43	0.90	0.003	0.65
	Boldness	0.46 ± 0.15	0.37	0.82	0.008	
Survival ~ sociability + island/year + sex + sociability × year/island	Year	−0.72 ± 0.17	0.51	0.98	<0.001	0.48
Survival ~ exploration + island/year + sex + exploration × year/island	Year	−0.54 ± 0.14	0.49	0.94	0.002	0.71
	Exploration	0.27 ± 0.14	0.19	0.41	0.083	
	Year × exploration	−0.50 ± 0.13	0.49	0.94	0.002	
	*Exploration 2012*	−0.55 ± 0.32				
	*Exploration 2013*	0.76 ± 0.23				

The personality trait domain variables (boldness, sociability and exploration) are based on individual scores from behavioural tests (see “Methods”). The final general linear models presented here were selected via backwards stepwise removal of non-significant terms. To ease interpretation of data, slopes for exploration within the interaction with year/island are given for each island/year separately. Note that maximum survival was set to 60 days and that this can result in conservative slope estimates. Animals that were lost or had an unknown fate were excluded from the statistical analysis. *df* = 1 in all cases

**Fig. 2:**
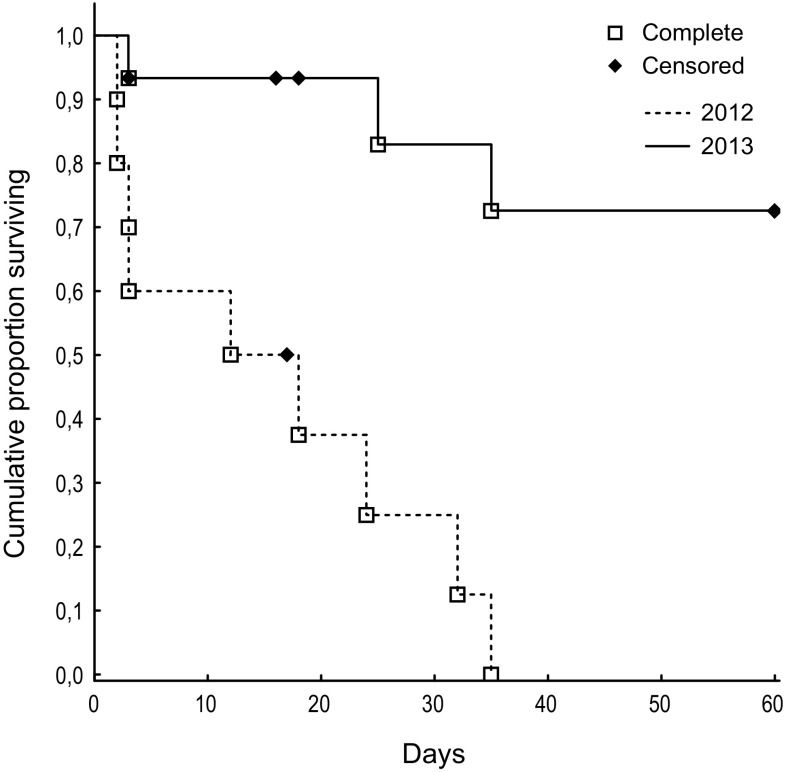
The cumulative proportion of surviving European mink post-release in Estonia during 2012 on Saaremaa and 2013 on Hiiumaa based on multiple groups Kaplan-Meier survival analysis with year as grouping variable. Animals that survived throughout the radio-tracking period have been censored as well as animals that were lost

In 2012, the mean survival time was 15 days and the cumulative survival for 60 days was 0 % (Fig. 2). Nine of the ten released animals died within 35 days after release, approximately half of them within the first three days after release. One animal was lost. Predation was the cause of death in seven cases, starvation in one case and one animal was hit by a car (Table 1). In 2013, the mean survival was 48 days and the cumulative survival for 60 days was 73 % (Fig. 2). Three of the fifteen released animals died within 35 days after release, one of them within the first 3 days after release. The cause of death was predation in all cases. Seven animals survived to the end of the 60 days of radio-tracking. Four animals were lost. The fate of one animal was labelled as unknown although a mortality signal, which persisted throughout the radio-tracking period, was retrieved from an underground drainage tunnel (Table 1). The tunnel could not be accessed, and as we could not rule out the possibility that the animal had not died but dropped the collar in the tunnel all analyses were run both with and without it. The inclusion/exclusion of this individual did not affect the outcome of the general linear model results. All statistical results, including the survival analysis, are presented without this individual. However, for illustration purposes it has been included in Fig. 1.

Overall, predation was the main cause of death in both years, making up 83 % of the total mortality. In 2012, many of the individuals killed by predators were fully or partially consumed, whilst none of the predator-killed individuals were eaten in 2013. The identified predators were red fox (*Vulpes vulpes*), domestic dog (*Canis familiaris*), pine marten (*Martes martes*) and domestic cat (*Felis catus*). In some cases the predator could not be identified.

## Discussion

In this study, we have found empirical evidence showing that survival in reintroduced European mink was related to the personality trait domains of boldness and exploration. Survival was positively related to boldness in both years, whereas exploration had a negative impact in 2012 and positive in 2013. The difference in the relationship between survival and exploration between the years in contrast to boldness, that did not vary, suggests a more complex relationship between survival and animal personality than previously shown. This result is of significance both in species conservation and to understand selection of personality. That the relationship between survival and exploration differed between the years and islands also supports the hypothesis that spatiotemporal variation in conditions might maintain personality over evolutionary time. A prerequisite for this assumption is that different personality types thrive under different conditions, so that selection alternates between favouring different personality types (e.g. Wolf et al. 2007; Wolf and Weissing 2010). In contrast, the relationship between survival and boldness was positive in both years and sociability had, as expected, no impact on survival. This suggests that a different mechanism might maintain different personality trait domains. Alternatively, the same mechanism, in this case fluctuating selection pressures, could be acting on all personality trait domains but on different fitness components, i.e. survival or reproduction. We suggest that the relationship between survival and personality, as well as the evolutionary background to personality, are complex subjects which need more attention in both empirical and experimental studies. It may also be of special importance to consider personality aspects in species conservation.

The mechanisms behind the relationship between survival and personality are largely unknown, and should be investigated in future studies. For example, although movement has been shown to be unrelated to survival in European mink, it has been suggested that movement data could reveal more information if analysed on a finer scale (Harrington et al. 2014). Personality-correlated variation in behavioural plasticity is also likely to be of interest. Different personality types have been shown to display different levels of behavioural plasticity in their strategies to cope with risky, novel and/or changing stimuli and conditions. More specifically, individuals in the high end of behavioural continuums such as boldness, exploration and aggression, tend to form lasting routines quickly while opposite personality types adapt continuously to the environment (e.g. Benus et al. 1987; Rodriguez-Prieto et al. 2010; Herborn et al. 2014).

While days survived was related to personality on both islands/years, and connected to spatiotemporal conditions via exploration, spatiotemporal conditions in themselves were also important for survival. Although predation was the main cause of death on both islands/years, no animal survived on Saaremaa throughout the radio-tracking period. There are several possible explanations for the difference in survival between islands/years. For example, both islands had been carefully assessed and found to contain suitable European mink habitat with adequate abundance of prey, but weather can affect productivity, and thus create fluctuations in food availability. The relative abundance and local distribution of different prey species could hence vary on a short temporal scale. This could affect both survival rates, and the success of different personality types, as these might have different coping strategies. The weather did indeed differ between the islands and years, with 2012 being wetter and colder than 2013. The summer mean temperature and rainfall/day was 15.8 °C and 2.58 mm, respectively, on Saaremaa in 2012 and 17.2 °C and 1.56 mm, respectively, on Hiiumaa in 2013. In addition, differences between the islands in densities of predators could affect the level of intraguild predation and competition. However, as Saaremaa has higher densities of red fox while Hiiumaa has higher densities of pine martens (Maran, pers. obs.), any impacts are difficult to assess.

Previous studies have shown that bold individuals generally have shorter life spans than shyer individuals (Smith and Blumstein 2008), and higher levels of boldness have been related to higher activity and feeding rates in the presence of predators. Furthermore, there are also more signs of predation attempts on bold individuals compared to shy (Carter et al. 2010). In contrast, we found that the personality trait domain assessed as boldness in European mink (see Haage et al. 2013) was positively related to days survived. This highlights the potential hazard of generalising between species, especially in a relatively new field of science where more empirical data are needed.

In contrast to previous studies on survival in reintroduced European mink (Maran et al. 2009) we did not detect a sex difference in survival. We, therefore, suggest that sex influences on survival are mediated by personality type, as males in general are bolder and more explorative than females (Haage et al. 2013). A potentially varying composition of personality types among the test animals in the two studies could, hence, explain the difference in results. This is especially likely since personality type was the main criteria used to select test animals in this study.

It is important to improve species conservation methods, e.g. in reintroduction programmes, to enhance the success rates, but also to increase the welfare of released individuals as mortality is considered to be a welfare issue (Harrington et al. 2013). Our findings support the suggestion that personality aspects should be included in conservation efforts (Watters and Meehan 2007). Different strategies need to be evaluated before any recommendations can be made. Still, examples of potential strategies include selecting animals for release based on their personality types, or anti-predator training of vulnerable personality types. In particular, as predation is the major cause of death for many reintroduced animals. However, selecting animals with specific personality types for release imposes a risk of decreasing genetic variation in the reintroduced population as personality is at least partially heritable (e.g. van Oers et al. 2005). Antipredator training could also be problematic as it can be costly and technically challenging (Griffin et al. 2000). It could also be argued that because personality is stable over time and/or contexts, any influence of personality on survival could not be removed via training. However, although personality is partially heritable it is also shaped during ontogeny (Groothuis and Carere 2005). Furthermore, personality types can be related to different rates of behavioural plasticity (e.g. Benus et al. 1987; Rodriguez-Prieto et al. 2010; Herborn et al. 2014). Hence training could be a viable option during ontogeny and also if a sensitive personality type simply needs more time to adapt to wild conditions, including predator cues.

By empirically and experimentally testing the influence of several personality trait domains on survival, we have found evidence that personality can explain survival patterns in reintroduced European mink. Our results also indicate that variation in spatiotemporal conditions might be one of the mechanisms maintaining personality in European mink over time, but also that there are differences between personality trait domains. Finally, our findings highlight that personality can be an important tool in conservation biology.

## Electronic supplementary material

Below is the link to the electronic supplementary material.

Supplementary material 1 (PDF 13 kb)

Supplementary material 2 (DOCX 16 kb)
