# Supplementary figures and images for: The influence of spatiotemporal conditions and personality on survival in reintroductions–evolutionary implications
Source: Oecologia. 2016 Oct 8;183(1):45–56. doi: 10.1007/s00442-016-3740-0 (PMC5239807; doi:10.1007/s00442-016-3740-0)

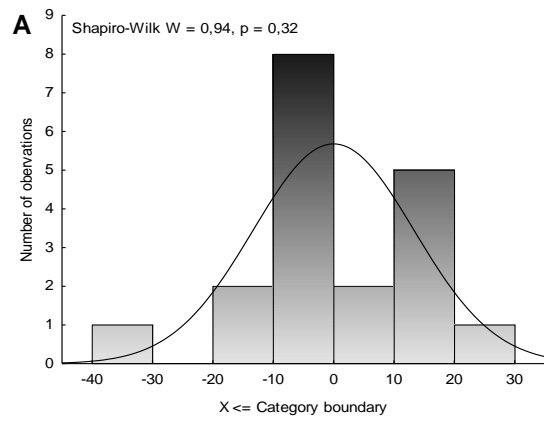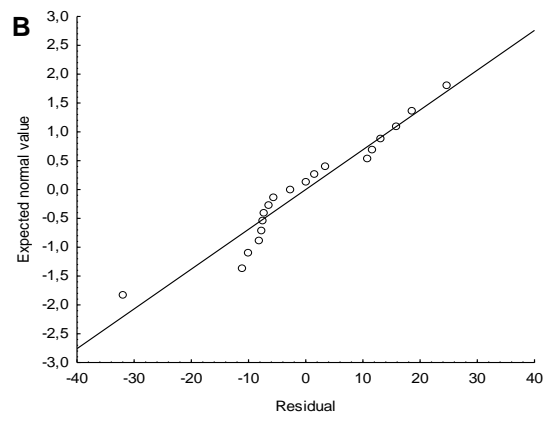

Supplement: Supplementary file 1 — Supplementary material 1 (PDF 13 kb) [file 442_2016_3740_MOESM1_ESM.pdf]
