# Supplementary material for: The influence of spatiotemporal conditions and personality on survival in reintroductions–evolutionary implications
Source: Oecologia. 2016 Oct 8;183(1):45–56. doi: 10.1007/s00442-016-3740-0 (PMC5239807; doi:10.1007/s00442-016-3740-0)
Supplement: Supplementary file 2 — Supplementary material 2 (DOCX 16 kb) [file 442_2016_3740_MOESM2_ESM.docx]

**Appendix 2** Global general linear models on the effect of personality and release year/island on post-release survival (days survived) in radio-tracked European mink (N = 19) released on the Estonian islands Saaremaa in 2012 and Hiiumaa in 2013. Animals that were lost or had an unknown fate were excluded from the statistical analysis. Df = 1 in all cases.

| **Global model** | **Variable** | **Beta (β) ± SE** | **Partial eta-squared** | **Power** | **p** | **Adjusted R^2^** |
| --- | --- | --- | --- | --- | --- | --- |
| Survival ~ boldness + island/year + sex + boldness*island/year | Year | -0.52 ± 0.17 | 0.41 | 0.82 | 0.008 | 0.61 |
|  | Sex | 0.072 ± 0.16 | 0.015 | 0.071 | 0.65 |  |
|  | Boldness | 0.44 ± 0.19 | 0.27 | 0.56 | 0.039 |  |
|  | Year*Boldness | 0.005 ± 0.17 | <0.001 | 0.050 | 0.98 |  |
| Survival ~ sociability + island/year + sex + sociability*island/year | Year | -0.71 ± 0.18 | 0.53 | 0.96 | 0.001 | 0.51 |
|  | Sex | 0.33 ± 0.19 | 0.18 | 0.36 | 0.11 |  |
|  | Sociability | -0.26 ± 0.24 | 0.081 | 0.18 | 0.29 |  |
|  | Year*Sociability | -0.058 ± 0.21 | 0.005 | 0.057 | 0.79 |  |
| Survival ~ exploration + island/year + sex + exploration*island/year | Year | -0.54 ± 0.14 | 0.50 | 0.94 | 0.002 | 0.71 |
|  | Sex | 0.15 ± 0.13 | 0.086 | 0.19 | 0.27 |  |
|  | Exploration | 0.22 ± 0.15 | 0.13 | 0.28 | 0.16 |  |
|  | Year*Exploration | -0.50 ± 0.13 | 0.50 | 0.94 | 0.002 |  |
